# A Novel Function for SNAP29 (Synaptosomal-Associated Protein of 29 kDa) in Mast Cell Phagocytosis

**DOI:** 10.1371/journal.pone.0049886

**Published:** 2012-11-21

**Authors:** Jordan Wesolowski, Vernon Caldwell, Fabienne Paumet

**Affiliations:** Department of Microbiology and Immunology, Thomas Jefferson University, Philadelphia, Pennsylvania, United States of America; Institute of Molecular and Cell Biology, Singapore

## Abstract

Mast cells play a critical role in the innate immune response to bacterial infection. They internalize and kill a variety of bacteria and process antigen for presentation to T cells via MHC molecules. Although mast cell phagocytosis appears to play a significant role during bacterial infection, little is known about the proteins involved in its regulation. In this study, we demonstrate that the SNARE protein SNAP29 is involved in mast cell phagocytosis. SNAP29 is localized in the endocytic pathway and is transiently recruited to *Escherichia coli* (*E. coli)*-containing phagosomes. Interestingly, overexpression of SNAP29 significantly increases the internalization and killing of *E. coli*, while it does not affect mast cell exocytosis of inflammatory mediators. To our knowledge, these data are the first to demonstrate a novel function of SNAP29 in mast cell phagocytosis and have implications in protection against bacterial infection.

## Introduction

Mast cells are well known for their key role in orchestrating the allergic response through the release of pro-inflammatory mediators following FcεRI aggregation, a process called degranulation [1]. However, evidence has also demonstrated a crucial role for mast cells in the innate immune response. Mast cells express Toll-like receptors 2 and 4, which allow them to recognize bacteria [2]. Stimulation through Toll-like receptors results in the secretion of pro-inflammatory cytokines, which participate in neutrophil and dendritic cell recruitment to the site of infection [3]. Furthermore, mast cells are able to internalize bacteria, and present bacterial antigen to CD8+T cells via MHC class I molecules [4]. Mast cells are prominent in areas of the host-environment interface such as the skin and mucosae [5]. As such, mast cells are situated at the major entry points for pathogens, which makes them one of the first phagocytic cells that bacteria may encounter.

Mast cells internalize bacteria using different mechanisms. For example, they internalize *E. coli* via caveolae structures [6], and *Mycobacterium tuberculosis* through cholesterol-rich microdomains [7]. Following their internalization, the bacteria-containing phagosomes undergo a maturation process whereby they ultimately fuse with the lysosomal compartments [8]. Similar to macrophages, the bactericidal activity of mast cells relies on oxidative bursts and acidification of phagocytic vacuoles [9]. Surprisingly, although phagocytosis is important for controlling bacterial infection, very little is known about the molecular mechanisms that regulate this pathway in mast cells.

In professional phagocytic cells such as macrophages, the process of phagocytosis is mediated by SNARE proteins (soluble N-ethylmaleimide-sensitive factor attachment protein receptor), which drive specific membrane fusion [8], [10], [11], [12]. These proteins, present on the surface of intracellular compartments, interact to form a stable complex, bringing together apposing membranes and triggering fusion [13], [14], [15]. SNAREs are classified depending on their location, either on vesicles (v-SNAREs) or target membranes (t-SNAREs). Whereas the v-SNARE is a single protein, the t-SNARE is composed of three subunits, one heavy chain and two light chains. In macrophages, the SNARE proteins Syntaxin7, Syntaxin13, VAMP7 and VAMP3 have been shown to control phagocytosis [11], [12], [16]. However, in mast cells no SNARE proteins have been implicated in the phagocytic process.

Previous studies using different model systems have described SNAP29, a member of the SNAP sub-family, as essential in a number of pathways including endocytosis, exocytosis and recycling [17], [18], [19], [20]. Here, we investigated the role of SNAP29 in mast cells and demonstrate that SNAP29 is involved in phagocytosis of *E. coli*. In particular, SNAP29 appears to play a major role in the killing process, during which SNAP29-positive endosomes relocate to *E. coli* phagosomes. Finally, we demonstrate that SNAP29 functions specifically in phagocytosis and is not involved in FcεRI-induced degranulation.

## Materials and Methods

### Cells and *E. coli* Bacterial Strain

The rat basophilic leukemia (RBL-2H3) cell line [21] was purchased from ATCC (CRL-2256). RBL-2H3 cells were maintained in complete DMEM (DMEM supplemented with 10% fetal bovine serum, 100 units/ml penicillin and 100 µg/ml streptomycin) as previously described [22]. Bone marrow-derived mast cells (BMMCs) were differentiated as described [23]. Briefly, bone marrow was obtained from C57BL/6 mice by flushing the femurs. The bone marrow cells were cultured for 4 weeks in RPMI 1640 supplemented with 10% fetal bovine serum, 4 mM glutamine, 1 mM sodium pyruvate, 100 units/ml penicillin, 100 µg/ml streptomycin, 1 mM nonessential amino acids (Invitrogen), 25 mM HEPES, 50 mM β-mercaptoethanol, and 30 µg/ml of recombinant mouse IL-3 (Shenandoah Biotechnology). The mast cell population was greater than 90% pure as determined by FcεRI and c-kit expression using flow cytometry. This study was approved by the Thomas Jefferson University Institutional Animal Care and Use Committee (IACUC). The *E. coli* strain [BL21 (DE3)-Invitrogen] transformed with a mCherry expression vector (kind gift from Dr. R. Tsien) was used for all assays.

### Antibodies

Anti-SNAP23, -SNAP29, -VAMP8, and -Actin rabbit polyclonal Abs were from Sigma-Aldrich. Anti-Rab4 and –Rab5 mouse monoclonal Abs were kind gifts from Dr. J. Keen (Thomas Jefferson University). Anti-VAMP8 mouse monoclonal Ab was from Santa Cruz Biotechnology. Anti-Syntaxin6 mouse monoclonal Ab was purchased from AbCam and the anti-GM130 monoclonal Ab was purchased from BD Transduction Labs. Peroxidase-coupled anti-rabbit Ab was obtained from GE Healthcare. AlexaFluor488 goat anti-rabbit Ab, AlexaFluor594 goat anti-mouse Ab and Hoechst was purchased from Invitrogen.

### DNA Manipulation and Plasmid Constructs

Standard PCR and ligation techniques were performed throughout. All PCR reactions were done with pfu turbo polymerase (Fisher). All other DNA modifying enzymes were from New England Biolabs. The *E. coli* strain DH5α (Invitrogen) was used for standard cloning. Plasmids encoding SNAP23-GFP and SNAP29-GFP were constructed by PCR amplification and ligation into the pEGFP-N3 vector (Clontech). The oligonucleotides used to clone SNAP23-GFP into the pEGFP-N3 vector (Clontech) are FO171: CGGAATTCTGCCACCATGGATGATCTATCACCAGAA (forward) and FO172: TTATGGATCCGCTGTCAATGAGTTTCTT (reverse). SNAP29-GFP was cloned using the oligonucleotides FO169: CGGAATTCTGCCACCATGTCAGCTTACCCTAAGAGG (forward) and FO170: TATGGATCCGAGTTGTCGAACTTTTCT (reverse). The empty pEGFP-N3 vector was used as a control.

### RBL-2H3 Cell Transfection

AMAXA nucleofector technology (Germany) was used to transfect RBL-2H3 cells. The procedure to establish stable populations expressing SNAP23-GFP, SNAP29-GFP and GFP is described in [22]. Briefly, 10^6^ cells were nucleofected in solution V (AMAXA) with 1 µg of either pEGFP-N3-*SNAP23* vector, pEGFP-N3-*SNAP29* vector or the pEGFP-N3 vector (control), using the program T-030. The transfected cells were then plated in 10 cm tissue culture plates for 3–5 days in complete DMEM before selection in 1.5 mg/ml G418. The media was changed every 5 days. As stable transfected populations survived, they were expanded. Stable populations were analyzed for GFP expression with a Nikon confocal microscope using a 60×oil-immersion objective, and frozen in freezing medium (10% FBS, 10% DMSO, 80% DMEM). Stably transfected populations were maintained with 1 mg/ml G418 in DMEM.

### Cell Fractionation

2×10^6^ cells/ml of RBL-2H3 mast cells or bone marrow-derived mast cells were resuspended in fractionation buffer (250 mM sucrose, 20 mM HEPES, 10 mM NaCl, 1.5 mM MgCl_2_, 1 mM EDTA, 1 mM EGTA, 1 mM DTT pH 7.4) containing complete EDTA-free protease inhibitor tablets (Roche). The cells were then disrupted by sonication. The homogenate was obtained by spinning the sonicated cells at 8,000 rpm for 5 min. To separate the cytosol from the membrane, the homogenate was centrifuged at 41,000 rpm for 1 h. The membrane pellet was washed in fractionation buffer to remove all cytosolic contaminants and centrifuged again at 41,000 rpm for 1 h. The membrane fraction was resuspended in the same volume of fractionation buffer as the cytosolic fraction. The homogenates and fractions were then solubilized with SDS at a final concentration of 1%. Equal volumes of the homogenate and the cytosolic and membrane fractions were analyzed by Western blotting.

### Gel Electrophoresis and Western Blotting

Proteins were separated on 10% Bis-Tris Gels (Invitrogen). Samples were transferred to a PVDF membrane (BioExpress) at 180 mA for 80 min. The membranes were subsequently blocked with 5% milk in wash buffer (25 mM Tris, 250 mM NaCl, 0.1% Tween-20, pH 7.6) and probed with primary antibodies as mentioned in Figure legends. The membranes were then washed and incubated with anti-rabbit secondary antibody (GE Healthcare). After additional washes, the membranes were revealed with ECL (GE Healthcare).

### Immunofluorescence Microscopy

The immunofluorescence experiments were conducted as described [22]. Briefly, RBL-2H3 cells were grown overnight on glass coverslips at a density of 5×10^4^ cells per coverslip. To assess protein localization during phagocytosis, *E. coli* was added at a multiplicity of infection (MOI) of 1,200 to the coverslips and spun at 1,000 rpm for 10 min. The coverslips were incubated at 4°C for 1 h to allow bacteria to adhere to the cells. They were then transferred to 37°C for an additional 1 h, 2.5 h or 4 h. At each time point, the cells were washed with cold PBS and fixed with 2% paraformaldehyde (PFA) for 30 min at 4°C. Next, the coverslips were washed with cold PBS and permeabilized in permeabilization buffer (20% Goat serum, 5 mg/ml BSA, 0.1% saponin in PBS, pH 7.4) for 30 min at 4°C. The cells were then blocked for 1 h at 4°C with blocking buffer (20% goat serum, 5 mg/ml BSA in PBS, pH 7.4). Primary antibodies were incubated on coverslips for 1 h at room temperature. Cells were labeled with the Abs as described in Figure legends. The coverslips were washed thoroughly with blocking buffer and incubated with AlexaFluor488 goat anti-rabbit or AlexaFluor594 goat anti-mouse secondary Abs. The nucleus was labeled with 1 µg/ml Hoechst (Invitrogen). The coverslips were then washed and mounted on glass slides with ProLong Anti-Fade reagent (Invitrogen). Bone marrow-derived mast cells were infected and labeled in suspension at a concentration of 1×10^6^ cells/ml. The staining protocol is similar to RBL-2H3 except that each step was followed by centrifugation at 500×g for 5 min before resuspending the cell pellet. To assess the intracellular localization of endogenous SNAP29, resting RBL-2H3 were fixed with 2% PFA for 30 min at 4°C and stained as described above. Confocal images were acquired using a Carl Zeiss LSM 510 UV META inverted confocal microscope with a Plan-Apo 63×oil immersion lens at room temperature and Zeiss AIM 4.2 SP1 software. For TIRF, cells were fixed and labeled as described above. TIRF microscopy was conducted using an Andor/Nikon TiE inverted microscope with PFS for image stability control with a 100×oil immersion TIRF lens at room temperature and MetaMorph v7.6.5 software. Image analysis, including 3D reconstruction, was performed using ImageJ (NIH). Phagosomes were reconstructed in 3D from 0.3 µm Z-sections taken on the Zeiss confocal microscope.

### Gentamicin Protection Assay

RBL-2H3 cells were seeded onto 6-well plates at a density of 5×10^5^ cells per well and incubated at 37°C overnight in complete DMEM. BMMCs were seeded in 24-well plates at a density of 1×10^6^ cells per well in complete medium and incubated at 37°C. *E. coli* was grown to an optical density of 0.5–1, washed and resuspended in DMEM without antibiotics. Control cells were fixed with 2% PFA. *E. coli* was added to the cells at a MOI of 250, spun at 1,000 rpm for 10 min at 4°C and incubated at 37°C for an additional 2 h to allow for internalization. After several washes, 100 µg/ml gentamicin for RBL-2H3 or 200 µg/ml for BMMCs was added for 1 h to kill extracellular bacteria. The cells were rinsed to remove gentamicin and replaced with DMEM without antibiotics for the remainder of the assay (t = 0). The infected cells were further incubated at 37°C for 1 h, 2 h, and 24 h to assess the killing rate. To determine the number of extracellular bacteria, the antibiotic-free cell culture medium was collected at different time points, centrifuged, and plated on LB agar at 37°C. To quantify intracellular bacteria, RBL-2H3 cells and BMMCs were collected at different time points, lysed with 0.5% Triton X-100 and plated on agar at 37°C. Colonies were enumerated 24 h after plating.

### β-hexosaminidase Assay

The assay was conducted as described in [22]. Briefly, 5×10^4^ RBL-2H3 cells per well were plated in a 96-well plate. The cells were then washed in Tyrode’s buffer (135 mM NaCl, 5 mM KCl, 5.61 mM D-glucose, 10 mM HEPES pH 7.3, 1.8 mM CaCl_2_, 1 mM MgCl_2_, 0.5 mg/ml BSA). For FcεRI stimulation, the cells were sensitized with 100 ng/ml of anti-DNP IgE (Sigma) for 2 h at 37°C. The cells were then stimulated with 200 µl of 1 ng/ml DNP-BSA (Sigma) in Tyrode’s buffer for 15, 30 and 60 min at 37°C. The unstimulated and 100% wells received only Tyrode’s buffer. At each time point, 25 µl of supernatant was collected for analysis. The total intracellular β-hexosaminidase content was determined by lysing the 100% wells with 7 µl of 20% Triton X-100. To measure the level of β-hexosaminidase in the supernatant, 50 µl of 1.3 mg/ml poly-N-acetylglucosamine (Sigma) was added to 25 µl of supernatant and incubated at 37°C for 90 min. The enzymatic reaction was stopped with 150 µl of Glycine buffer (0.2 mM Glycine, pH 10.7). The absorbance was read at 405 nm using a BioTeck plate reader.

### Statistical Evaluation

The Mann-Whitney *U* test was used to compare the mean values of maximal release between the control and the different transfectants. Significance was assumed at p values<0.05.

## Results

### Mast Cells Express SNAP29

**Figure 1:**
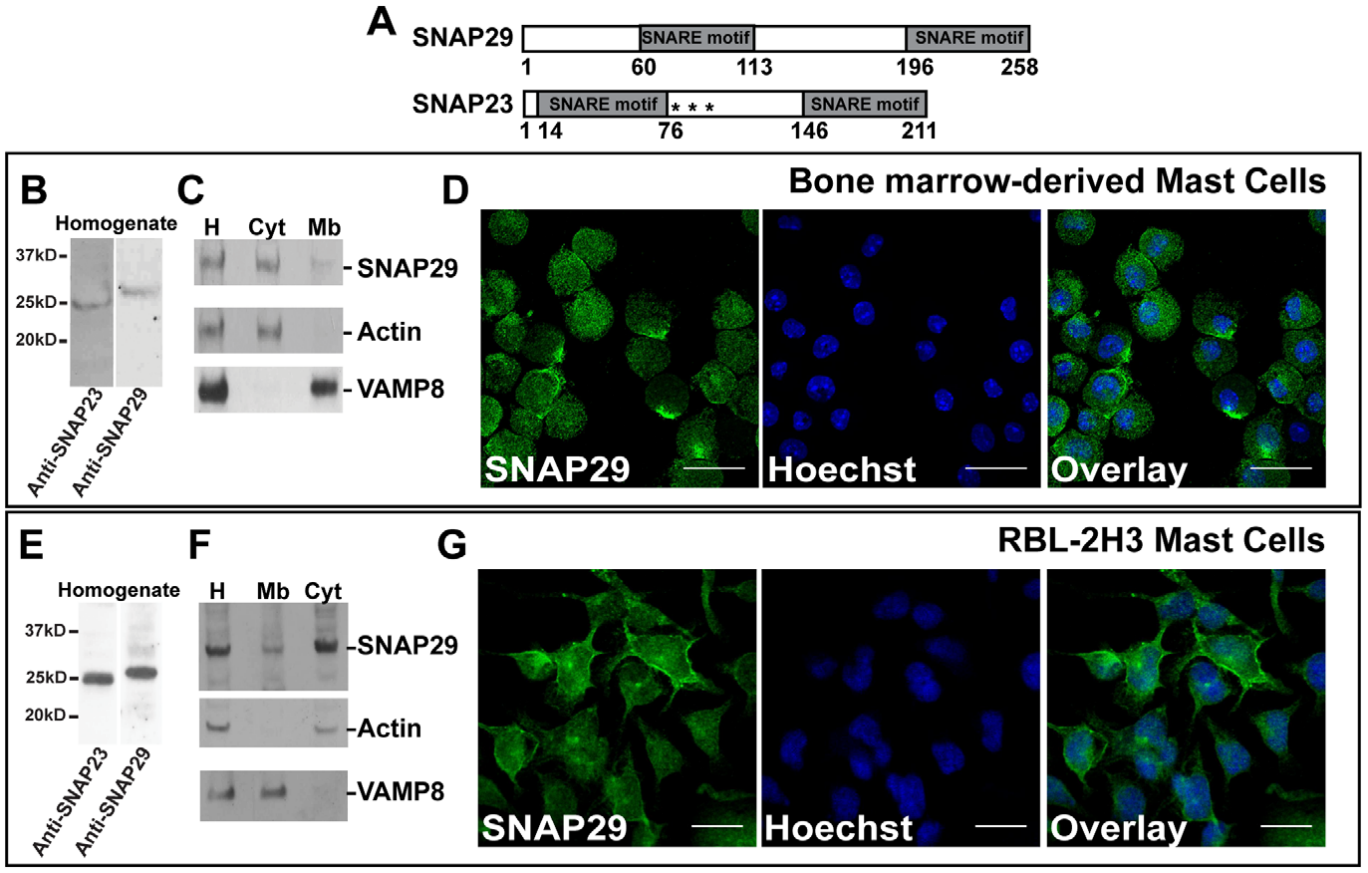
SNAP29 is distributed in both cytosolic and membrane fractions in resting mast cells. (A) Schematic of SNAP29 and SNAP23 SNARE proteins. Both proteins contain two SNARE motifs. The SNARE motifs in SNAP29 are in positions 60–113 and 196–258, and in positions 14–76 and 146–211 for SNAP23. Unlike SNAP23, SNAP29 does not contain palmitoylation sites (indicated by the asterisks for SNAP23). (B) Bone marrow derived mast cell (BMMC) homogenate was tested for SNAP29 expression by Western blot using an anti-SNAP29 Ab. As a control, SNAP23 expression was confirmed using an anti-SNAP23 Ab. 7×10^5^ cells were loaded in each lane. (C) BMMC homogenate (H) was fractionated into both cytosol (Cyt) and membrane (Mb) fractions. Fractions were solubilized with 1% SDS prior to loading. Equal volumes of each fraction were tested for SNAP29 expression by Western Blot using an anti-SNAP29 Ab. Cell fractionation was controlled using anti-actin (marker for cytosol) and anti-VAMP8 (marker for membranes) Abs. (D) Resting BMMCs were fixed, permeabilized, labeled with anti-SNAP29/anti-Rabbit AlexaFluor488 Ab and analyzed by confocal microscopy (Green). The nucleus was labeled with Hoechst (Blue). The overlay is presented on the right. Scale bar = 20 µm. (E) RBL-2H3 homogenate was prepared and immunoblotted with both anti-SNAP23 and anti-SNAP29 Abs. 3.5×10^5^ cells were loaded in each lane. (F) RBL-2H3 were fractionated and immunoblotted as described in *C*. (G) Fixed RBL-2H3 were labeled for SNAP29 as described in *D*. Scale bar = 20 µm. Each experiment is representative of n = 3.

SNAP29 is a member of the SNAP25 family of SNARE proteins and contains two SNARE motifs separated by a linker region (Figure 1A). Unlike SNAP23, SNAP29 does not possess a lipidic anchor (Figure 1A asterisks for SNAP23) in its linker region, which suggests that SNAP29 can only associate with membranes by interacting with membrane-bound proteins. Although SNAP29 has been shown to be expressed in numerous cell types, it has never been identified in innate immune cells [17], [18], [19], [20]. Here, we investigated the expression of SNAP29 in mast cells, which play a critical role in orchestrating the immune response against pathogens. To do so, bone marrow-derived mast cells (BMMCs) as well as RBL-2H3 mast cells were used. RBL-2H3 is a well-characterized rat mast cell line that expresses the high affinity IgE receptor FcεRI, and can be stimulated with IgE/allergen complexes [21]. Importantly, RBL-2H3 and BMMCs have also been shown to internalize and kill bacteria [7].

First, the expression of SNAP29 in mast cells was determined using western blot. As illustrated in Figure 1B and 1E, both BMMCs and RBL-2H3 express SNAP29 as a single species of ∼30 kDa that migrates at a higher molecular weight than its SNAP23 homologue.

The localization of SNAP29 in resting mast cells was investigated using immunofluorescence microscopy. To this end, permeabilized BMMCs and RBL-2H3 were stained with an anti-SNAP29 Ab and counter-stained with a nuclear marker. Endogenous SNAP29 was then analyzed by confocal microscopy. As shown in Figure 1D and 1G, SNAP29 appears to be both cytosolic and membrane-bound. In particular, SNAP29 seems to be associated with the plasma membrane and small vesicles. The distribution of SNAP29 (cytosol versus membrane) in resting mast cells was further investigated using cell fractionation in both BMMCs and RBL-2H3. As illustrated in Figure 1C and 1F (upper panels), SNAP29 was indeed found in both the cytosolic and membrane fractions. This distribution is in agreement with previous reports that showed a similar partitioning of SNAP29 in COS and NRK cells [24], [25]. As mentioned previously, SNAP29 has neither a transmembrane domain nor a lipid anchor (Figure 1A) [24], supporting the cytosolic localization of the protein. SNAP29 membrane staining however is likely due to its ability to bind one or multiple membrane proteins that remain to be identified.

### SNAP29 is Located on the Plasma Membrane and Endocytic Compartments

**Figure 2:**
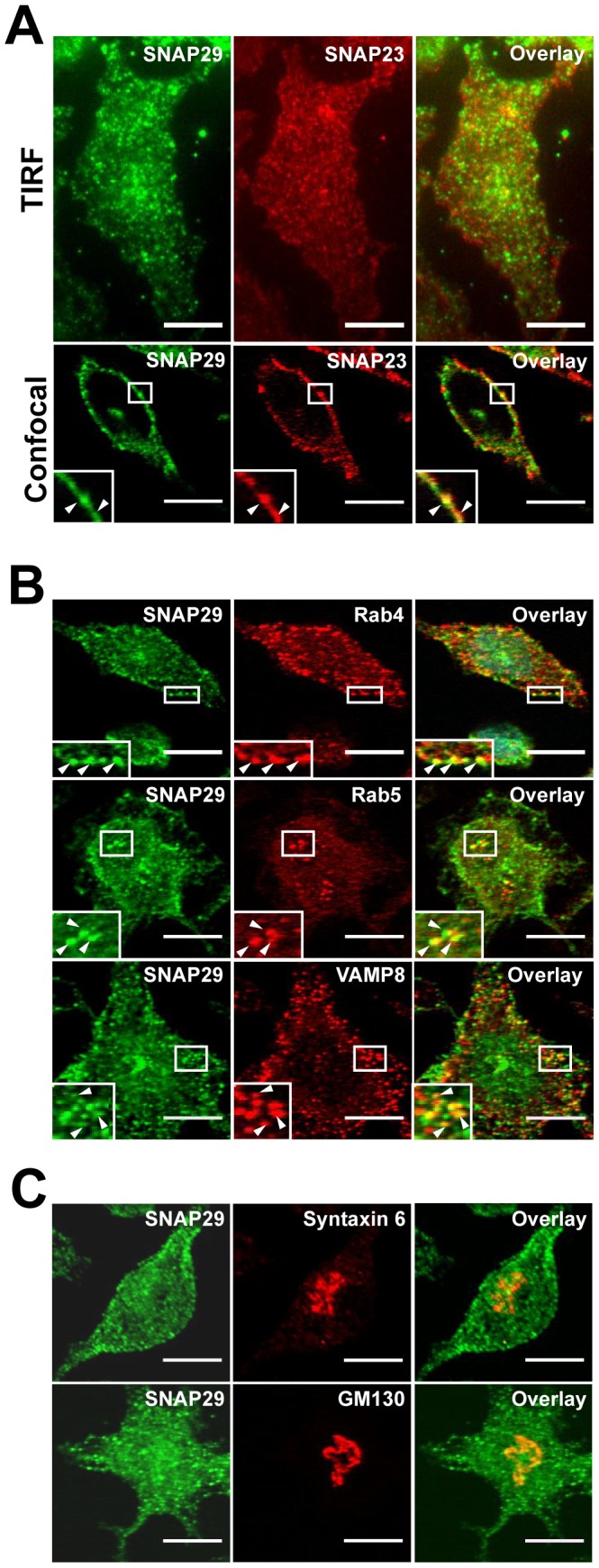
SNAP29 is localized on the plasma membrane and in endocytic compartments. (A) RBL-2H3 cells were fixed, permeabilized, labeled with anti-SNAP29/anti-Rabbit AlexaFluor488 Ab and anti-SNAP23/anti-Mouse AlexaFluor594 Ab and analyzed by TIRF microscopy (top panels) or confocal microscopy (lower panels). To visualize the plasma membrane in confocal, a high focal plane was used. Thus, cytoplasmic SNAP29 is not visible. (B) RBL-2H3 cells were fixed, permeabilized, labeled with anti-SNAP29/anti-Rabbit AlexaFluor488 Abs and anti-Rab4 (top panels), anti-Rab5 (middle panels), or anti-VAMP8/anti-Mouse AlexaFluor594 Abs (lower panels) and analyzed by confocal microscopy. (C) RBL-2H3 cells were fixed, permeabilized, labeled with anti-SNAP29/anti-Rabbit AlexaFluor488 Abs and anti-Syntaxin6 (top panels), or anti-GM130/anti-Mouse AlexaFluor594 Abs (lower panels) and analyzed by confocal microscopy. All scale bars = 10 µm. Arrowheads indicate areas of colocalization.

Initial confocal microscopy analysis showed that SNAP29 labeled both the plasma membrane and small intracellular vesicles (Figure 1D and 1G). We then decided to identify the intracellular compartments on which SNAP29 resides using immunofluorescence TIRF and confocal microscopy. SNAP23 was used as a marker for the plasma membrane [22], [26]. SNAP29 localization on the plasma membrane was first assessed using TIRF microscopy (Figure 2A, top panels). TIRF microscopy allows for the visualization of events at the interface between the plasma membrane and the coverslip with a penetration depth of 100 nm. RBL-2H3 cells were fixed, permeabilized and co-labeled with anti-SNAP29 and anti-SNAP23 Abs. The cells were then focused in TIRF using SNAP23 to locate the plasma membrane, followed by analysis of SNAP29 labeling. As shown in Figure 2A (top panels), SNAP29 and SNAP23 both stained RBL-2H3 cells in the same focal plane suggesting that SNAP29 is located on the plasma membrane. To validate this result, RBL-2H3 cells co-labeled for SNAP29 and SNAP23 were then analyzed by confocal microscopy. As shown in Figure 2A (lower panels), SNAP29 staining is similar to SNAP23 labeling. Collectively, these microscopy data demonstrate that SNAP29 is localized on the plasma membrane.

Next, we investigated the identity of the vesicular pool of SNAP29. To do so, SNAP29 was co-stained with different markers of intracellular compartments in resting RBL-2H3 cells before being analyzed using confocal microscopy. As shown in Figure 2B, SNAP29 partially localizes with Rab4 (top panels), a marker of recycling/sorting endosomes as well as with Rab5-containing early endosomes (middle panels) suggesting that SNAP29 may function as part of the early endocytic pathway. We then determined whether SNAP29 was also localized in the late endocytic pathway. The SNARE VAMP8 was used as a marker for late endosomes/lysosomes [27], [28]. SNAP29 partially localized to VAMP8 positive compartments (Figure 2B lower panels). Interestingly, it has been shown that the majority of VAMP8 is present on secretory granules [22], however there was no colocalization of SNAP29 with secretory granules (data not shown). This is consistent with the fact that the colocalization of SNAP29 with VAMP8 is minimal. Finally, SNAP29 localization in the Golgi was investigated. Previous studies have shown that SNAP29 interacts with the t-SNARE Syntaxin6, a Golgi marker [25], [29]. Here, SNAP29 was co-labeled with anti-Syntaxin6 or anti-GM130 Abs. Syntaxin6 marks the trans-Golgi while GM130 is found in the cis-Golgi. As shown in Figure 2C, no clear colocalization between SNAP29 and Syntaxin6 (top panels) or GM130 (lower panels) was observed. Altogether, these data demonstrate that SNAP29 is located on the plasma membrane and in the endocytic pathway, most significantly with early endosomes, while being absent from the Golgi.

### SNAP29 is not Involved in Mast Cell Secretion of Inflammatory Mediators

**Figure 3:**
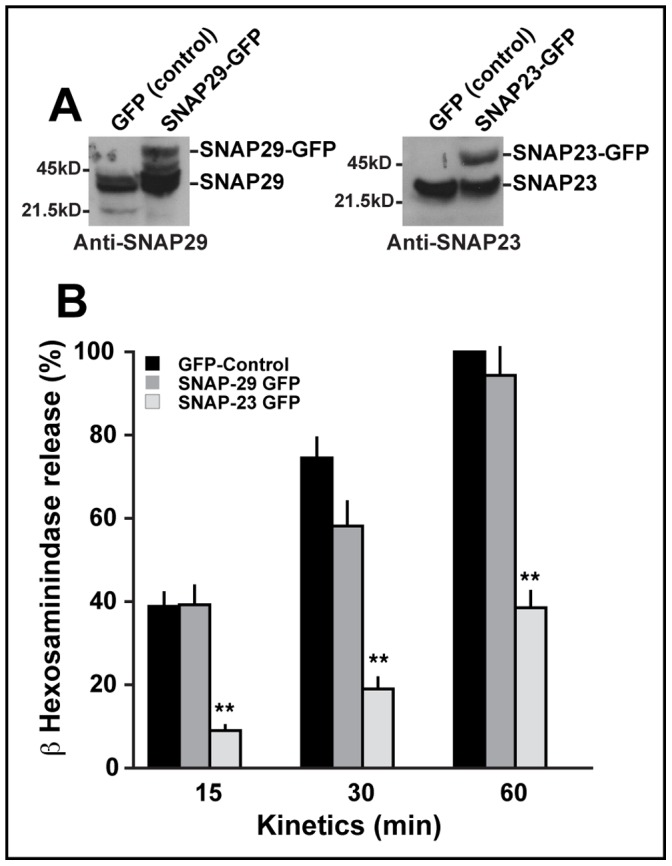
SNAP29 is not involved in mast cell degranulation. (A) RBL-2H3 cells were stably transfected with SNAP29-GFP, SNAP23-GFP or GFP. Homogenates for each transfected population were immunoblotted with anti-SNAP29 and anti-SNAP23 Abs. 1.25×10^6^ cells were loaded in each lane. For each transfected population, two bands corresponding to the endogenous SNARE (lower band) and the SNARE-GFP (higher band) were observed. (B) Transfected RBL-2H3 cells were sensitized with anti-DNP IgE for 2 h, stimulated with DNP-BSA for 15, 30, and 60 min and β-hexosaminidase release was assayed. The data shown are the mean ± SD of five independent experiments each performed in triplicate. The asterisks denote a significant difference (p<0.01).

Since a pool of SNAP29 is found on the plasma membrane, we investigated whether SNAP29 plays a role in mast cell degranulation. To do so, we specifically used the RBL-2H3 cell line since it allowed us to generate cell populations stably overexpressing SNAP29-GFP, which would not have been possible in primary mast cells. In parallel, two RBL-2H3 cell populations stably expressing GFP (negative control) and SNAP23-GFP were established. The level of expression for both SNAP29-GFP and SNAP23-GFP is shown in Figure 3A. Overexpression of SNARE proteins is a common method to determine whether a protein is involved in a specific pathway, which is indicated by a disturbance in the given process (increase or decrease of transport) [22], [30], [31]. Overexpression is particularly useful when functional redundancy is present in the system [32], [33], [34].

FcεRI-dependent degranulation was quantified for SNAP29-GFP expressing cells by measuring the kinetics of granular β-hexosaminidase secretion into the cell culture medium. Interestingly, overexpression of SNAP29-GFP does not significantly affect mast cell degranulation, compared to the GFP control (Figure 3B). In contrast, the overexpression of SNAP23-GFP drastically affects β-hexosaminidase secretion compared to the GFP control. At 15 min, the cells expressing SNAP23-GFP secreted only 8% of β-hexosaminidase compared to 39% for GFP. At 60 min, while the GFP control has attained its maximum secretion (100%), SNAP23-GFP expressing cells have only released 39% of β-hexosaminidase. These results confirm the involvement of SNAP23 in mast cell degranulation [26]. Although SNAP29 has been previously implicated in exocytosis in *C. elegans* [18], [35], our results show that in mast cells, despite its plasma membrane location, SNAP29 is not involved in the exocytosis of inflammatory mediators.

### SNAP29 Localizes to the Phagosome in *E.coli* Infected Mast Cells

The capacity of mast cells to internalize and kill bacteria is well recognized and underscores the important role of mast cells in the innate immune response against pathogens [3]. Since SNAP29 is not involved in mast cell degranulation and our data, as well as previous studies, place SNAP29 in the endocytic pathway [17], [18], [19], [20], we investigated whether SNAP29 played a role in mast cell phagocytosis.

**Figure 4:**
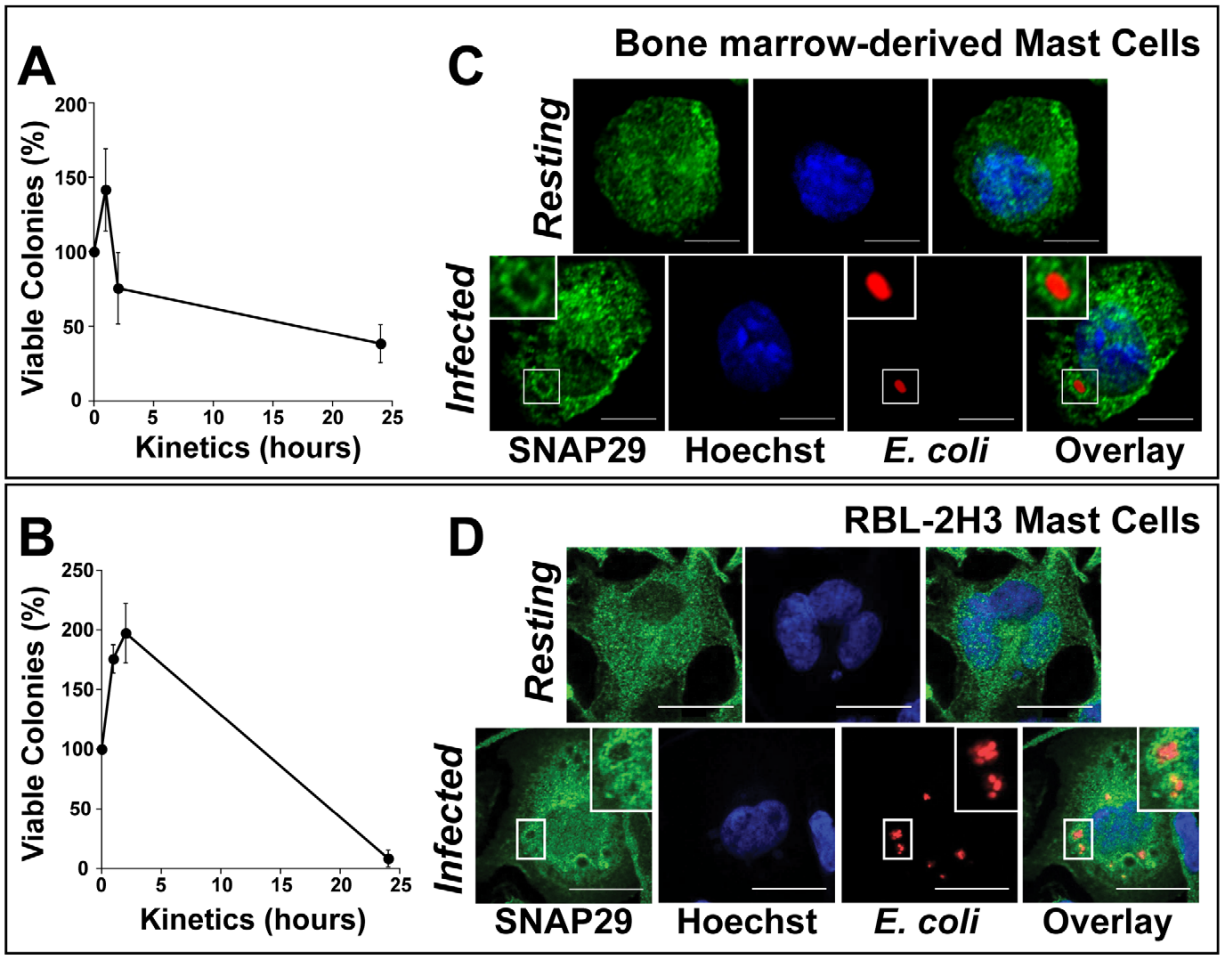
Mast cells kill *E. coli* while SNAP29 relocates to the phagosome. BMMCs (A) and RBL-2H3 cells (B) were infected with *E. coli* at a MOI of 250 for 2 h at 37°C. Extracellular bacteria were killed with gentamicin for 1 h. Cells were then washed and incubated with DMEM without antibiotics. The cells were incubated at 37°C for an additional hour, 2 h or 24 h. Cell lysates were serially diluted on agar plates at 37°C. Surviving colonies were counted 24 h later. Both graphs represent the mean ± SD of three independent experiments, each performed in duplicate. BMMCs (C) and RBL-2H3 (D) were infected with mCherry *E. coli* (red) for 1 h, fixed, permeabilized, and labeled with anti-SNAP29/anti-rabbit AlexaFluor488 Abs (Green). The nucleus was labeled with Hoechst (Blue). SNAP29 colocalization with *E. coli* was assessed using confocal microscopy. SNAP29 staining accumulates around *E. coli* 1 h post infection. The *inset* in each image shows an enlarged area of an *E. coli* phagosome. This experiment is representative of n = 3. Scale bar = 5 µm (C) and 20 µm (D).

First, we established the bactericidal capacity of both BMMCs and RBL-2H3 mast cells. To do so, BMMCs and RBL-2H3 were infected for 2 h with *E. coli* at a multiplicity of infection (MOI) of 250, followed by incubation for 1 h in the presence of gentamicin to kill extracellular bacteria. Then, the infected cells were collected at t = 0, 1 h, 2 h and 24 h, lysed, and plated on LB agar at 37°C to determine the number of surviving *E. coli*. Only internalized *E. coli* are protected from gentamicin and grow on agar. The number of internalized bacteria at 1 h, 2 h, and 24 h was compared to the number of internalized bacteria at t = 0 (arbitrarily defined as 100%) for each mast cell type. Both BMMCs and RBL-2H3 internalized and killed *E. coli* during a 24 h period (Figure 4A and B). Interestingly, during the first hours after internalization, we observed an increase in the number of colonies recovered in BMMCs and RBL-2H3. This may reflect slow killing kinetics in mast cells, which would allow *E. coli* to replicate inside the phagosome during the first hours before being killed. BMMCs appear to more efficiently kill *E. coli* than RBL-2H3 since we observed a significant decrease in viable colonies 2 h after internalization for BMMCs (142% at t = 1 h to 76% at t = 2 h). In RBL-2H3, this decrease is delayed and takes place one hour later. Twenty-four hours after infection, BMMCs killed greater than 60% of internalized *E. coli* (Figure 4A) and RBL-2H3 killed more than 80% (Figure 4B). This data confirms the killing capacity and bactericidal role of mast cells in both BMMCs and RBL-2H3 cells [9].

Both early and late endosomal compartments are involved in maturation of the phagosome prior to fusion with the degradative lysosomal compartments. Since SNAP29 is localized in the endocytic pathway in mast cells, we investigated whether SNAP29 was involved in the killing of *E. coli*. To do so, the intracellular location of SNAP29 was examined during *E. coli* phagocytosis. We anticipated that a relocation of SNAP29 towards the phagosomes containing *E. coli* would constitute an indication that SNAP29 is involved in the phagocytic process. BMMCs and RBL-2H3 were infected with *E. coli* constitutively expressing mCherry, a fluorescent marker. Bacteria internalization as well as the location of the endogenous SNAP29 was determined 1 h post-infection. As illustrated in Figure 4C, SNAP29 relocates to phagosomes that contain *E. coli* in BMMCs. Similarly, we observe a relocation of SNAP29 to the *E. coli* phagosomes in RBL-2H3 mast cells (Figure 4D). The fact that (i) SNAP29 relocates to phagosomes containing *E. coli* in both BMMC and RBL-2H3, in conjunction with (ii) the similar localization of SNAP29 in both resting mast cell types and (iii) the ability of both BMMCs and RBL-2H3 to kill *E. coli*, supports RBL-2H3 mast cells as a strong model for mast cell phagocytosis.

**Figure 5:**
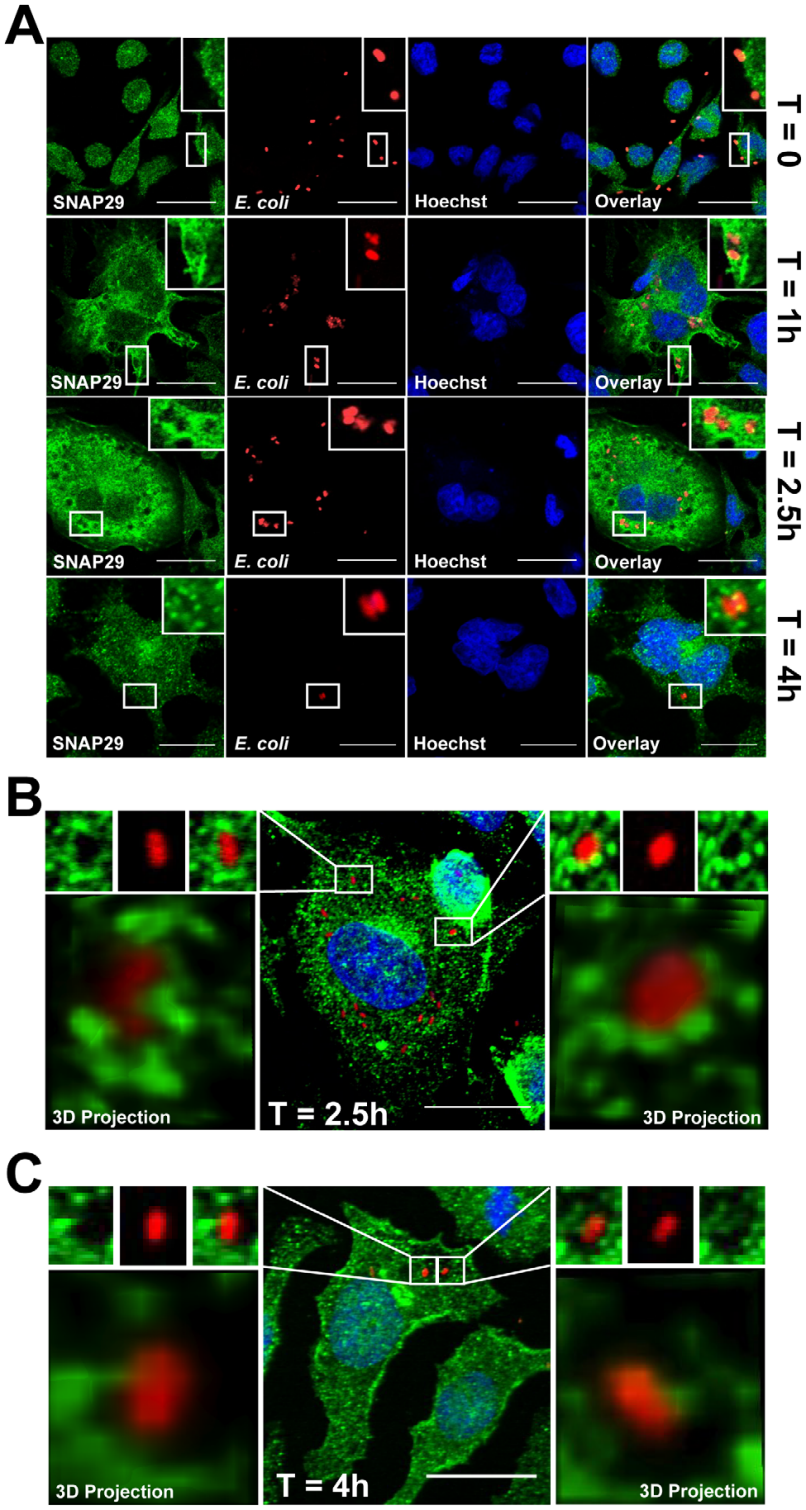
SNAP29 relocation to *E. coli* phagosomes in infected mast cells is transient. (A) RBL-2H3 cells were infected with *E. coli* expressing mCherry (red) for 0 h, 1 h, 2.5 h or 4 h. Cells were then fixed, permeabilized and labeled with anti-SNAP29/anti-rabbit AlexaFluor488 Abs (green). The nucleus was labeled with Hoechst (blue). SNAP29 localization was assessed using confocal microscopy. SNAP29 staining accumulates around *E. coli* phagosomes at t = 1 h and t = 2.5 h. The *inset* in each image shows an enlarged area of *E. coli* phagosomes. This experiment is representative of n = 3. Scale bars = 20 µm. (B and C) RBL-2H3 cells were infected with *E. coli* mCherry (red) for 2.5 h (B) or 4 h (C). Cells were fixed and stained as described in *A*. SNAP29 localization was assessed using confocal microscopy. Two different *E. coli* phagosomes are shown on the left and right of the center image. Z-sections of 0.3 µm were used to reconstruct each phagosome in 3D (shown below the enlarged phagosomes). SNAP29-positive endosomes associate with phagosomes at 2.5 h, but are not present 4 h post-infection. Scale bars = 20 µm.

To further characterize the involvement of SNAP29 in mast cell phagocytosis, we determined the kinetics of SNAP29 relocation in RBL-2H3 infected with *E. coli* at 1 h, 2.5 h, and 4 h post-infection. As illustrated in Figure 5A, at t = 0, while *E. coli* is bound to the mast cell surface, SNAP29 localization is identical to resting cells. As shown previously (Figure 4D), SNAP29 localization changes after 1 h at 37°C. At this time, SNAP29 appears to be enriched around *E. coli* phagosomes (Figure 5A t = 1 h), where it remains for a few hours (Figure 5A t = 2.5 h). Four hours post-infection, the location of SNAP29 is similar to its location in resting mast cells (Figure 5A t = 4 h), suggesting that the relocation of SNAP29 towards the phagosome is a transient event.

Interestingly, the relocation of SNAP29 around the phagosomes appears to be punctate in nature, rather than a continuous labeling of the phagosomal membrane, suggesting that SNAP29 may be present on small vesicles adjacent to the phagosomes. To investigate this possibility, *E. coli* phagosomes were further analyzed using confocal microscopy and 3D reconstruction. As illustrated in Figure 5B (center image and enlarged phagosomes), 3D reconstruction of phagosomes 2.5 h after infection reveals that SNAP29-positive endosomes are intimately interacting with the phagosome. In some cases, SNAP29-positive endosomes appear to be curving around the bacteria. However, at 4 h (Figure 5C) SNAP29-positive endosomes are not found close to the bacterium, which is consistent with our previous observations (Figure 4A t = 4 h). Altogether these results suggest that SNAP29-positive endosomes transiently interact with *E. coli* phagosomes during maturation.

### SNAP29 is Involved in Mast Cell Phagocytosis

**Figure 6:**
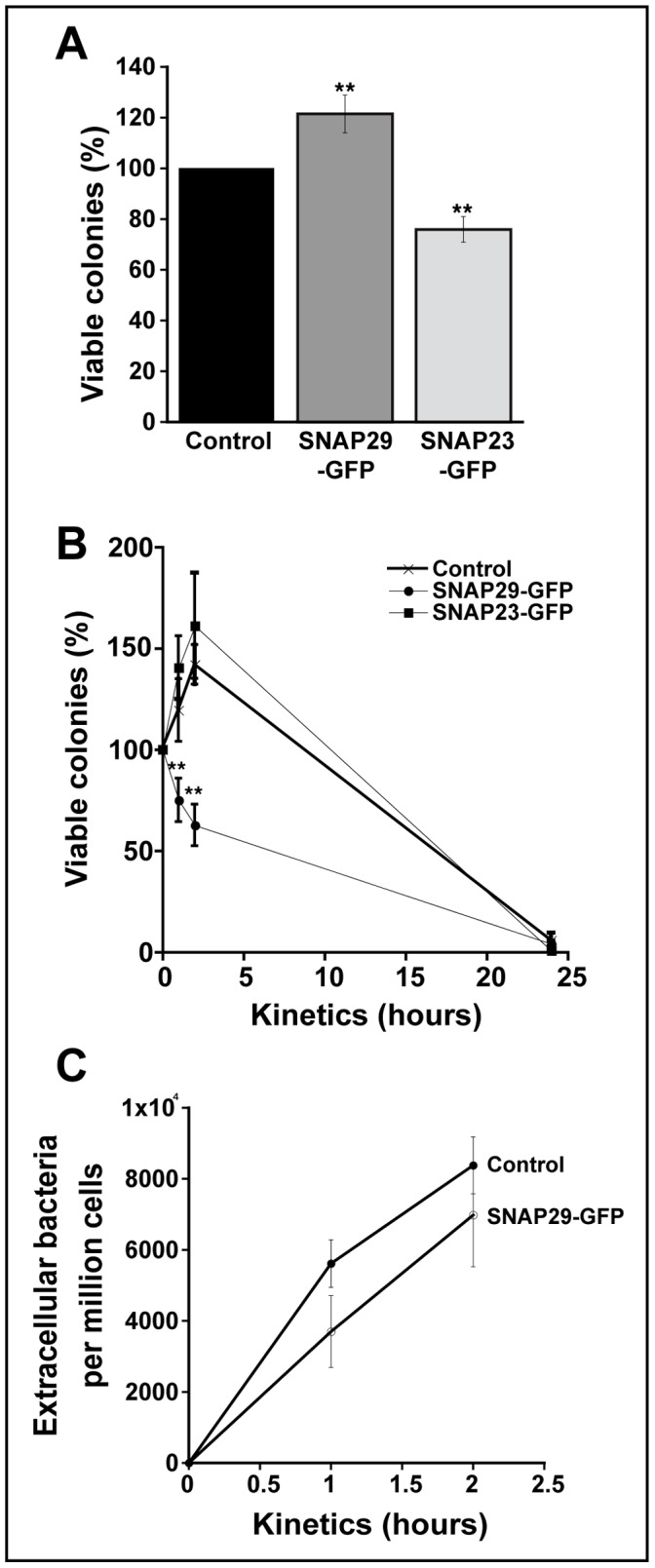
SNAP29 is involved in mast cell phagocytosis. (A) RBL-2H3 cells overexpressing GFP, SNAP29-GFP, or SNAP23-GFP were infected with *E. coli* at a MOI of 250 for 2 h at 37°C. Extracellular bacteria were killed with gentamicin for 1 h. Cells were then lysed and serially diluted on agar plates at 37°C. Surviving colonies were counted 24 h later. (B) Transfected RBL-2H3 cells were infected as described in *A*. Extracellular bacteria were killed with gentamicin for 1 h. The infected cells were then washed and incubated with DMEM without antibiotics. The cells were returned to 37°C for an additional hour, 2 h or 24 h. Cell lysates were serially diluted on agar plates at 37°C. Surviving colonies were counted 24 h later. (C) After transfected RBL-2H3 cells were infected with *E. coli* (MOI 250), the culture medium was collected, centrifuged to concentrate the bacteria and serially diluted on agar plates at 37°C. Colonies were counted 24 h later. All the graphs represent the mean ± SD of five independent experiments, each performed in triplicate. The asterisks denote a significant difference (p<0.01).

The relocation of SNAP29 towards *E. coli* phagosomes highlights its potential involvement in phagocytosis. To test this possibility, RBL-2H3 cells overexpressing SNAP29-GFP, SNAP23-GFP or GFP were tested in a series of phagocytic assays. First, the rate of *E. coli* internalization was quantified. To do so, transfected RBL-2H3 cells were infected with *E. coli* at a MOI of 250 for 2 h at 37°C and treated with gentamicin for 1 h at 37°C. This was considered t = 0. The infected cells were then lysed, and plated on LB agar at 37°C to determine the number of surviving *E. coli*. As illustrated in Figure 6A, the overexpression of SNAP23-GFP seems to slightly reduce internalization (80% versus 100%), whereas the overexpression of SNAP29-GFP increases the internalization of *E. coli* compared to the GFP control. Although the increase in internalization is limited (120% for SNAP29-GFP versus 100% for GFP), it is statistically significant and may reflect a function of the SNAP29 plasma membrane pool in phagocytosis. During phagocytosis, intracellular compartments fuse with the phagocytic cup to supply membrane for the formation of phagosomes [36]. A number of different compartments, namely recycling endosomes, late endosomes/lysosomes and secretory granules, have been shown to fuse with plasma membrane during phagocytosis [36]. In macrophages, recycling endosomes fuse with the plasma membrane at the level of the phagocytic cup in a SNAP23-dependent manner [36], [37]. The fact that both overexpressed SNAP29-GFP and SNAP23-GFP affect mast cell phagocytosis suggests that both of these SNAREs may participate in fusion events occurring at the plasma membrane during internalization in mast cells.

We then determined the impact of SNAP29-GFP overexpression on the killing capacity of mast cells. Cells overexpressing SNAP29-GFP, SNAP23-GFP and GFP were infected with *E. coli* and the number of surviving bacteria at 1 h, 2 h, and 24 h was compared to the number of internalized bacteria at t = 0 for each transfected population (arbitrarily defined as 100%). Normalizing the kinetics of each transfected population to its own t = 0 (100%) allowed us to compare the kinetics of these three populations despite differences in internalization. As shown in Figure 6B, the killing kinetics for both the GFP and the SNAP23-GFP cells are similar. Overall, both populations are able to eliminate the internalized *E. coli* in 24 h. Note that there is an increase in the number of colonies during the first 2 h, which is consistent with what was observed in non-transfected RBL-2H3. Interestingly, the SNAP29-GFP transfected cells show a remarkable decrease in the number of viable *E. coli* during the first 2 h (average of 150% viable colonies for both GFP and GFP-SNAP23 versus 60% for SNAP29-GFP at t = 2 h). This decrease is not due to recycling of the intracellular bacteria back to the extracellular medium since the number of extracellular bacteria measured for GFP-SNAP29 is not higher than the GFP control (Figure 6C). Collectively, these data suggest that SNAP29 is involved in phagocytosis.

## Discussion

Phagocytosis is an important process for the elimination of invading pathogens, foreign particles and dead cell bodies. During the course of this study, we confirmed the phagocytic function of mast cells using BMMCs and the rat RBL-2H3 mast cell line. We showed that mast cells not only internalize *E. coli*, but also kill these bacteria within 24 h. The killing of *E. coli* appears to be relatively slow, allowing *E. coli* to replicate during the first 2 h after internalization. The slow kinetics could be due to the fact that trafficking through the endocytic pathway to the lysosome may be slower in mast cells. In fact, the relative contribution of individual intracellular lytic systems appears to vary depending upon the role of the phagocytic cells. For instance, compared to macrophages and neutrophils, the pH of the phagosome in dendritic cells (DC) is much higher and does not decrease over time [38]. In these cells, the enzymatic content of the lysosome or lytic granules is also reduced and as a result, their potency is greatly diminished [39]. In addition, the acquisition of lysosomal markers in DCs is also remarkably slower compared to macrophages, highlighting the differences in lytic capacity of various cell types [40]. Thus, it is possible that, like dendritic cells, the killing capacity of mast cells may be differentially regulated compared to macrophages. This observation may reflect the multitude of processes that mast cells juggle during the immune response.

The slow killing capacity of mast cells may also be linked with the bacterial strain that is internalized. In macrophages for example, mechanisms of phagosome formation and maturation, and ultimately the killing of bacteria are highly dependent on the nature of the bacterium. Non-pathogenic bacteria are internalized and degraded within the phagolysosome [41]. On the other hand, pathogenic bacteria can not only avoid recognition [42] or induce their own internalization [43], but also corrupt the trafficking of their phagosome and subsequent fusion with the lysosome [44], [45]. In the case of mast cells, they respond to FimH+*E. coli* differently than to their FimH- counterparts [9]. FimH is a subunit of type I fimbriae expressed by enterobacteria, which promotes the binding of bacteria to mucosal surfaces. Mast cells appear four times more efficient at killing FimH+compared to FimH- *E. coli*, which may be due to the greater oxidative burst triggered by FimH+*E. coli* [9]. In addition to *E. coli*, mast cells respond to a variety of different bacteria [7], [46], [47], [48]. The intracellular mechanism by which mast cells differentially process bacteria is still unclear but it will be interesting to study the kinetics of bactericidal killing of other bacteria. Altogether, the differential trafficking of pathogenic and non-pathogenic bacteria highlights the complexity of the phagocytic pathway, which is not only dependent on the phagocytic cell type, but also on the strain of bacteria.

Elements of the phagocytic molecular machinery have been identified in professional phagocytes [11], [12], [16], [49]. Mast cells however, whose phagocytic function is now well established, have not been studied in this regard. Here we demonstrate that the SNARE protein SNAP29 is involved in phagocytosis. In resting mast cells, SNAP29 is distributed in both membrane and cytosolic fractions. Although it is unclear whether these pools have different functions, the distribution of SNAP29 in mast cells is similar to the distribution observed in other cell types [24], [25]. Using confocal microscopy, we demonstrated that the membrane-associated SNAP29 is present on the plasma membrane and on early endosomes. To our knowledge, SNAP29 has never been shown to associate with the plasma membrane. What could be the function of this plasma membrane pool of SNAP29? Here, we established that SNAP29, unlike its homologue SNAP23, is not involved in the exocytosis of inflammatory mediators in mast cells. However, we observed that the overexpression of SNAP29 and SNAP23 in mast cells affected the internalization of *E. coli*. Since nascent phagosomes are derived from the plasma membrane, SNAP29 and SNAP23 may be involved in the early process of phagosome formation. Sustained phagocytosis requires the continuous replacement of cell surface membrane from intracellular sources [50], [51], [52]. In macrophages, SNAP23 is involved in the fusion of recycling endosomes with phagocytic cup to supply additional membrane to the forming phagosome [37]. In mast cells, the plasma membrane pools of SNAP29 and SNAP23 may participate in the fusion events necessary to replenish the membrane stock that decreases during phagocytosis. Consistent with this possibility, SNAP29 is able to interact with Syntaxin4 [24], [53], a t-SNARE shown to be involved in mast cell exocytosis when bound to SNAP23 [22].

Because SNAP29 is not restricted to a specific compartment, unlike most SNARE proteins, it has been suggested that SNAP29 may be involved in multiple transport steps [24], [53]. In fact, SNAP29 has been implicated in a variety a pathways including endocytosis, recycling, and exocytosis [17], [18], [19], [20], [35], [54], [55], [56]. Consistent with this result, in mast cells, we observe partial, but significant colocalization with Rab4-positive recycling endosomes and Rab5-positive early endosomes, whereas the colocalization of SNAP29 with late endosomes/lysosomes is limited. Interestingly, SNAP29-positive endosomes transiently associate with *E. coli* phagosomes. 3D reconstructions of confocal Z-sections establish that SNAP29-positive endosomes intimately interact with the phagosomes at 2.5 h. Importantly, using a gentamicin protection assay, we discovered that SNAP29, unlike SNAP23, is involved in phagocytosis during which it appears to regulate the killing of the internalized *E. coli*. Although both SNAP23 and SNAP29 are potentially involved in the formation of nascent phagosomes (Figure 6A), SNAP29 is the only one involved in phagosomal maturation. The restricted plasma membrane location of SNAP23 likely prevents SNAP23 from being further involved in the process. This is supported by the absence of SNAP23 on the phagosomes (data not shown).

Altogether, this constitutes yet another function for SNAP29 in that it likely regulates the fusion of the phagosomes with the endocytic compartments, and ultimately the generation of the degradative phagolysosome.

In professional phagocytic cells various SNAREs have been implicated in the phagocytic process, including Syntaxin7, Syntaxin13, VAMP7 and VAMP8 [11], [12], [57]. Early during phagosomal maturation, Syntaxin13 appears to mediate the fusion of early endosomes with the phagosome, along with Syntaxin6 and Vti1a [11], [58]. Later, Syntaxin7, which is localized on the late endocytic compartments, interacts with the t-SNAREs Syntaxin8 and Vti1b, and the v-SNARE VAMP7 to trigger the formation of the phagolysosomes [59], [60], [61]. This process is highly regulated since VAMP8, another late endocytic SNARE has been shown to negatively regulate phagocytosis in dendritic cells, probably by competing with VAMP7 [57]. Although our functional data clearly establish the involvement of SNAP29 in mast cell phagocytosis, because we are using an overexpression strategy to study the role of SNAP29 (knock-down of SNAP29 expression using shRNA was unsuccessful in mast cells), it is difficult to conclude whether SNAP29 is positively or negatively regulating this process. Nevertheless, SNAP29 has been shown to bind a number of SNARE proteins *in vitro* including Syntaxin7, and Syntaxin13 [24], [53]. Since SNAP29 is mainly present on the endocytic compartments, it will be important to determine whether either or both these proteins form a fusogenic complex with SNAP29 in mast cells, which could potentially be involved in the early phagocytic process. Identifying cognate SNARE partners of SNAP29 will greatly contribute to understanding the function of SNAP29 in phagocytosis.

In summary, we show that SNAP29, a SNARE member of the SNAP sub-family, is involved in mast cells phagocytosis. This is a novel function for SNAP29 and it will be important to establish whether this role is conserved in other professional phagocytic cells such as macrophages and dendritic cells. Understanding the molecular machinery controlling phagocytosis in mast cells will be a crucial step towards evaluating the physiological implications of this process and its importance during bacterial infections. Furthermore, a complete identification of the fusogenic SNARE complexes involved in different intracellular trafficking events in mast cells, and evaluating the interplay between these SNAREs will be critical to understanding how this unique cell can juggle so many physiological processes necessary to orchestrate the innate immune response.
